# Severe Hypercalcemia in Burkitt Lymphoma Following Heart Transplant in an Elderly Male

**DOI:** 10.7759/cureus.84680

**Published:** 2025-05-23

**Authors:** Alyssa Kang, Shivani K Modi, Ashish Banjade, Justyna Kacarow, Sabin Tripathee

**Affiliations:** 1 Internal Medicine, Jefferson Einstein Healthcare Network, East Norriton, USA; 2 Internal Medicine, Einstein Medical Center Philadelphia, Norristown, USA; 3 Internal Medicine, Jefferson Einstein Montgomery Hospital, East Norriton, USA; 4 Internal Medicine, Jefferson Einstein Medical Center Montgomery, East Norriton, USA

**Keywords:** acute hypercalcemia, burkitt lymphoma, ebv reactivation, non-hodgkin's lymphoma, non-pth-related hypercalcemia of malignancy, post-cardiac transplant, post-transplant lymphoproliferative disorder, post-transplant malignancy, severe hypercalcemia, sporadic burkitt lymphoma

## Abstract

Burkitt lymphoma (BL) is a highly aggressive B-cell non-Hodgkin lymphoma (NHL) that rarely occurs as a post-transplant lymphoproliferative disorder (PTLD), especially in the elderly. We report a rare case of BL in a male in his 70s who developed BL several years following heart transplantation in the setting of chronic immunosuppression. He initially presented with signs and symptoms of severe hypercalcemia and retroperitoneal lymphadenopathy, which was biopsied to confirm the diagnosis. Treatment was initiated with rituximab and intrathecal methotrexate; however, his hospital course was complicated by a sigmoid microperforation due to a newly formed colonic fistula. This is an unusual case of Epstein-Barr virus (EBV) reactivation causing PTLD and underscores the importance of considering aggressive lymphomas in immunocompromised elderly patients following solid organ transplantation.

## Introduction

Burkitt lymphoma (BL) is an aggressive, mature B-cell lymphoma that is known to have a characteristic “starry-sky” appearance imparted by the scattered tingible body macrophages engulfing apoptotic debris of the tumor [1]. The disease is associated with Epstein-Barr virus (EBV) and human immunodeficiency virus (HIV). Although BL accounts for ∼40% of all childhood non-Hodgkin lymphoma (NHL), it represents <5% of lymphoma cases in adults [2]. BL post-transplant lymphoproliferative disorders (PTLD) are a rare subtype of post-transplant lymphoma representing less than 10% of adult PTLD [3]. PTLDs are a heterogeneous group of conditions that involve uncontrolled proliferation of lymphoid cells associated with extrinsic immunosuppression and EBV after solid organ or hematopoietic stem cell transplantation [4,5]. Severe hypercalcemia is an uncommon complication of BL that can clinically manifest as acute encephalopathy, nephrolithiasis, or gastrointestinal sequelae. This is a unique case of BL presenting as symptomatic hypercalcemia in an elderly male, several years after heart transplantation.

## Case presentation

**Figure 1:**
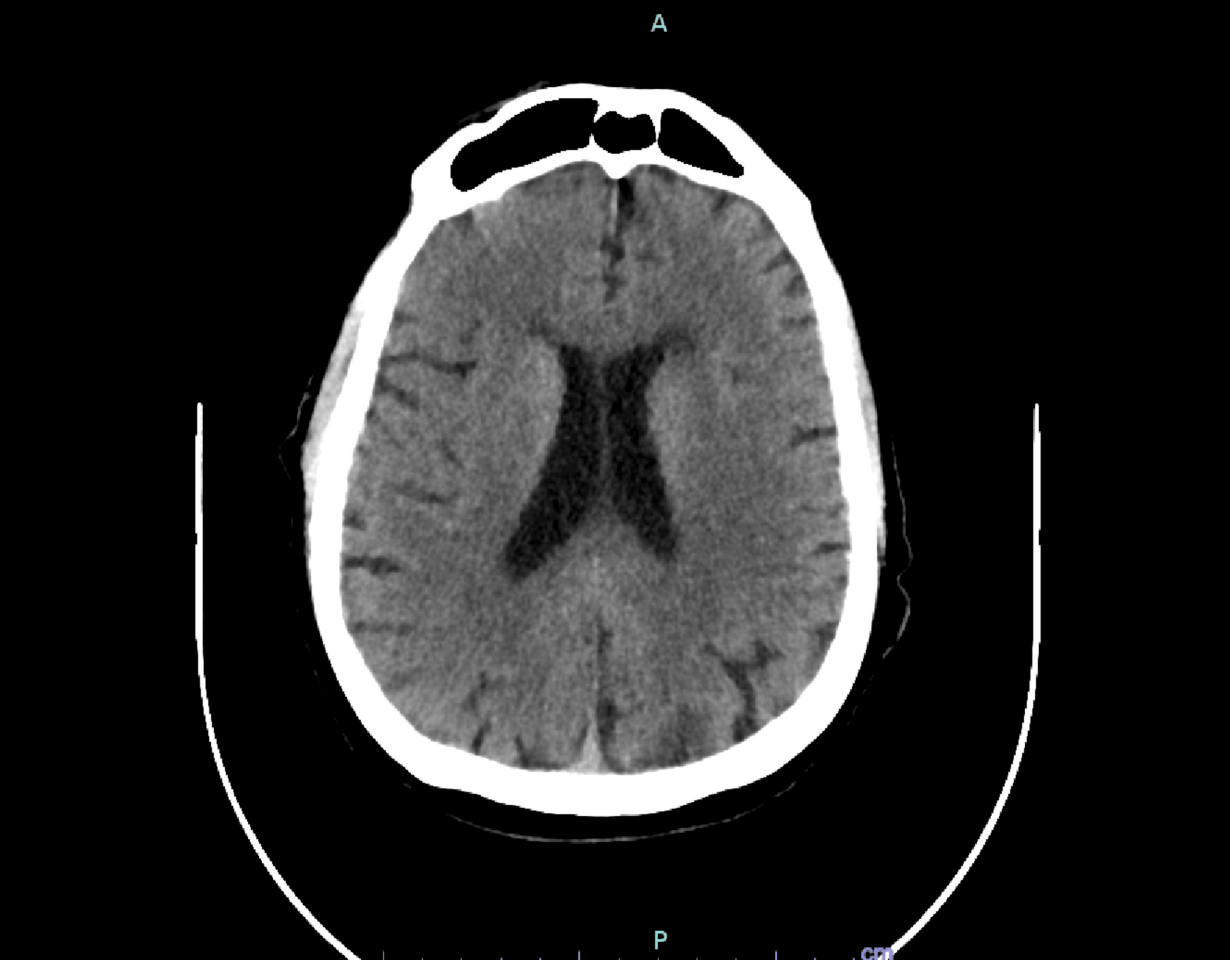
Computed tomography of the head showed no acute abnormalities

**Figure 2:**
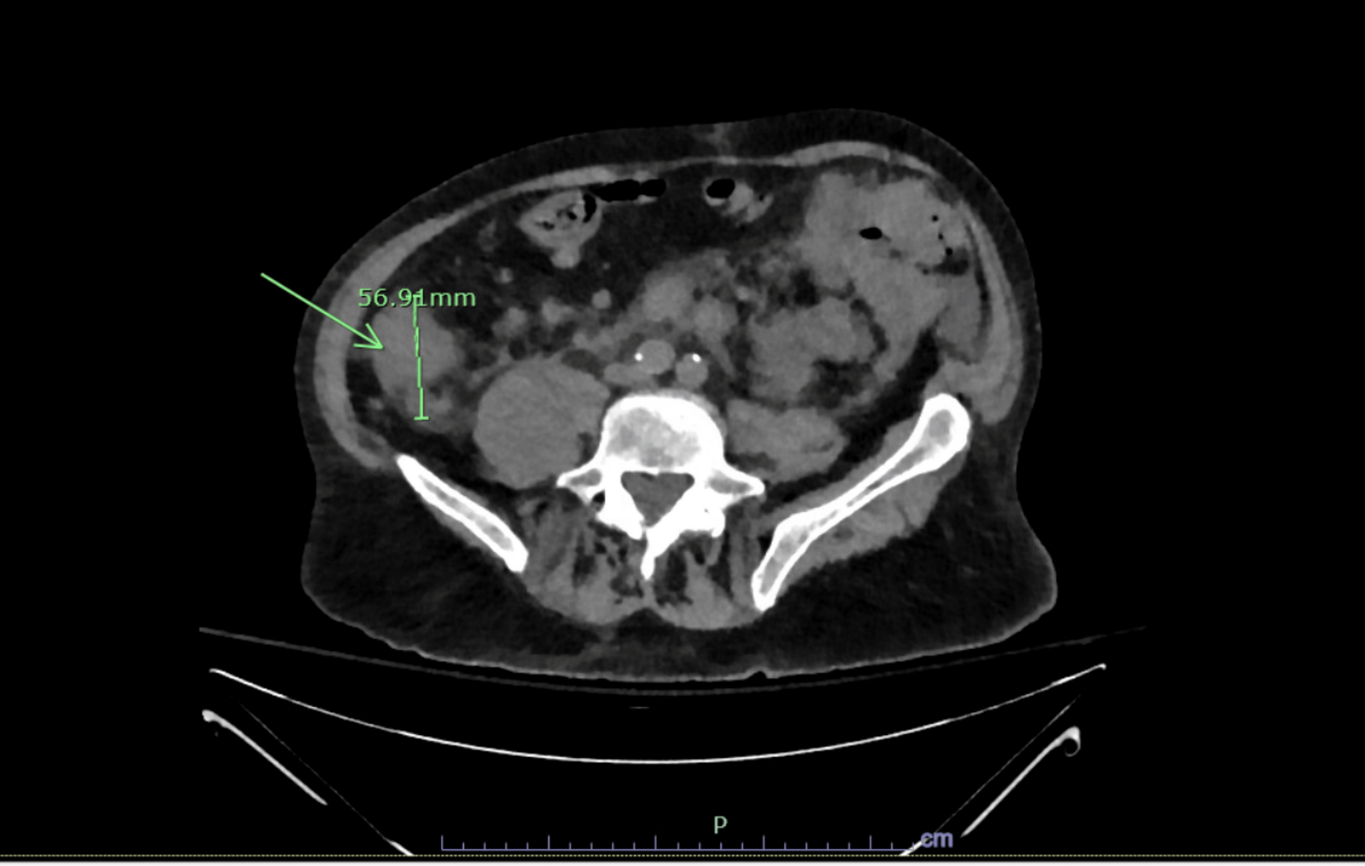
CTAP demonstrating a 5.7 cm retroperitoneal soft tissue implant CTAP, computed tomography of the abdomen and pelvis

Our patient was a male in his 70s with a history of anterior myocardial infarction complicated by cardiogenic shock requiring heart transplantation 22 years prior, as well as hypothyroidism and stage 3b chronic kidney disease, who presented to the hospital with altered mental status. He arrived in an agitated state, and there was a report from family members that he was not at his baseline mentation. On physical exam, he was alert and oriented to person and place but not to location or time. Cranial nerves II through XII were intact. Initial laboratory results were notable for a corrected calcium level of 16.7 mg/dL, undetectable parathyroid hormone, creatinine of 1.69 mg/dL (baseline 1.2 mg/dL), hemoglobin of 12.5 g/dL, and white blood cell count of 12.8 × 10³/mm³ (Table 1). Computed tomography (CT) brain was normal (Figure 1). He was treated with aggressive fluids, calcitonin, and zoledronic acid with appropriate improvement in mentation. CT of the abdomen and pelvis showed multiple retroperitoneal soft tissue implants and wall thickening in the proximal sigmoid colon, concerning lymphomatous involvement of the colon (Figure 2). A biopsy of one of the retroperitoneal implants revealed a B-cell population positive for CD20, CD10, c-MYC, and BCL-6, and negative for BCL-2 and CD30, with a Ki-67 proliferative index approaching 100%, along with scattered background CD3+ T-cells. Magnetic resonance imaging (MRI) of the brain showed no evidence of metastasis. A lumbar puncture was also performed and was normal. The patient was treated with rituximab and intrathecal methotrexate and subsequently transferred to a higher-level facility for further management.

**Table 1:** Laboratory data

Lab test	Value	Reference Range
WBC	12.8 x 1000/mm^3^	4 - 11
Hemoglobin	12.5 gm/dL	14 - 18
Creatinine	1.69 mg/dL	0.7 - 1.2
Calcium	16.7 mg/dL	8.4 - 10.3
PTHi	<4.0 pg/mL	9 - 73
Vitamin D 25-OH	80 ng/mL	30 - 100

## Discussion

PTLDs represent a significant complication in solid organ transplant recipients, with BL being one of the most aggressive forms. This case highlights the unique challenges in diagnosing and managing BL in the context of immunosuppression following transplantation. PTLDs occur in approximately 1-20% of transplant recipients, with incidence varying by organ type, intensity of immunosuppression, and EBV status [6]. BL represents only 2-3% of all PTLDs but carries a particularly poor prognosis due to its aggressive nature [7]. The median time to PTLD development is typically six months to one year post-transplantation, though late-onset cases like ours are increasingly recognized [8]. Risk factors include high-intensity immunosuppression, EBV mismatch (EBV-negative recipient and EBV-positive donor), and use of T-cell-depleting antibodies [9]. Our patient tested negative for HIV and developed BL despite adherence to immunosuppressive regimens, highlighting that even well-managed transplant patients remain at risk. EBV reactivation was inferred due to the absence of other identifiable causes. 

BL in transplant recipients typically develops through EBV-driven B-cell proliferation in the setting of impaired T-cell surveillance [10]. The characteristic t(8;14) translocation, which places the c-MYC oncogene under the control of immunoglobulin heavy chain enhancers, was identified in our patient's tumor cells, consistent with classical BL. This genetic alteration leads to constitutive expression of c-MYC, driving rapid cell proliferation with a doubling time of 24-48 hours [11]. Immunosuppressive agents, particularly calcineurin inhibitors, impair T-cell function and cytokine production, compromising immune surveillance against EBV-infected B cells and emerging malignant clones [12]. This creates a permissive environment for lymphomagenesis, as evidenced in our patient who developed BL while on immunosuppression. 

Diagnosis of BL in transplant recipients presents unique challenges. Clinical presentations may be atypical, with extranodal involvement being common [13]. In our case, the patient presented with symptomatic hypercalcemia that initially gave way to a broad differential including malignancy, familial hypocalciuric hypercalcemia, and primary hyperparathyroidism. PTH was undetectable, ruling out primary hyperparathyroidism from our differential. 1,25-dihydroxycholecalciferol was also within normal limits. The exact mechanism by which hypercalcemia manifests from NHL is unclear, but it is generally not a calcitriol or PTHrp-mediated process. Recent data demonstrate that calcitriol elevation has an association with worse outcomes of NHL, implying that this may be a marker in high-grade lymphoma. 

In our patient, the "starry sky" histopathological pattern and high Ki-67 proliferation index (>95%) were key to establishing the diagnosis, along with confirmation of c-MYC rearrangement by FISH analysis [14]. PET-CT imaging demonstrated extensive lymphoma involving the retroperitoneum, pelvis, spleen, colon, mesentery, and muscles, consistent with the hypermetabolic nature of BL. However, interpretation of PET findings in transplant recipients requires caution due to potential false positives from infection or inflammation [15]. 

Management of BL in transplant recipients requires a delicate balance between effective lymphoma therapy and maintenance of graft function. Reduction of immunosuppression (RI) is typically the first step in PTLD management, but for high-grade lymphomas like BL, RI alone is insufficient [16]. Our patient received tacrolimus and dexamethasone while initiating systemic therapy. 

Intensive chemotherapy regimens such as R-CODOX-M/IVAC or DA-EPOCH-R have shown efficacy in BL but carry significant toxicity risks in transplant recipients [17]. Our patient received cyclophosphamide with dose modifications to account for lung toxicity. Following multiple interdisciplinary discussions between the patient, oncology, and infectious disease, a decision was made to not pursue more aggressive treatment given the patient's older age and personal preference. He also refused to be tested for EBV. Prophylaxis against tumor lysis syndrome with allopurinol was administered and was essential due to the high proliferation rate of BL cells [18]. Rituximab, which targets CD20-positive B cells, has improved outcomes in PTLD and was incorporated into our patient's treatment plan [19]. Central nervous system (CNS) prophylaxis with intrathecal chemotherapy was administered due to the high risk of CNS involvement in BL [20]. 

Historically, BL in transplant recipients has carried a poor prognosis, with reported five-year overall survival rates of 25-35%. However, recent advances in supportive care and targeted therapies have improved outcomes. Our patient received his first chemotherapy session with rituximab and intrathecal methotrexate at an out-of-network tertiary-level hospital. There, he developed acute abdominal pain, and a repeat CT imaging study showed extraluminal gas adjacent to the sigmoid colon concerning microperforation in the setting of extensive lymphadenopathy, but no progression was noted. Two days later, he experienced hematochezia but was denied surgical intervention. His treatment course was complicated by acute abdominal pain, and a CT scan demonstrated a fistula between the sigmoid colon and masses along the left paracolic gutter with signs of microperforation of the sigmoid colon. He was initially discharged to comfort care with intravenous meropenem but improved clinically over the course of two months. He pursued treatment again and received a second cycle of chemotherapy with rituximab, cyclophosphamide, and etoposide. Since the second treatment, he has required two hospital admissions for community-acquired pneumonia related to drug-induced pneumonitis. He remains on long-term dexamethasone and tacrolimus therapy and is otherwise in stable condition.

## Conclusions

This case highlights the challenges associated with the diagnosis and management of PTLD precipitated by EBV reactivation. Factors associated with eventual favorable outcomes included early diagnosis and preserved organ function. Challenges in our case included the patient’s older age and chronic immunosuppressed state, which predisposed him to respiratory infections and gastrointestinal complications following therapy. This was managed through close follow-up with his transplant team and appropriate adjustments to his immunosuppressant regimen.

## References

[1] Picarsic J, Jaffe R, Mazariegos G, Webber SA, Ellis D, Green MD, Reyes-Múgica M (2011). Post-transplant Burkitt lymphoma is a more aggressive and distinct form of post-transplant lymphoproliferative disorder. Cancer.

[2] Bishop PC, Rao VK, Wilson WH (2000). Burkitt's lymphoma: molecular pathogenesis and treatment. Cancer Invest.

[3] Zimmermann H, Reinke P, Neuhaus R (2012). Burkitt post-transplantation lymphoma in adult solid organ transplant recipients: sequential immunochemotherapy with rituximab (R) followed by cyclophosphamide, doxorubicin, vincristine, and prednisone (CHOP) or R-CHOP is safe and effective in an analysis of 8 patients. Cancer.

[4] Dharnidharka V, Webster A, Martinez O (2016). Post-transplant lymphoproliferative disorders. Nat Rev Dis Primers.

[5] Dierickx D, Habermann TM (2018). Post-transplantation lymphoproliferative disorders in adults. N Engl J Med.

[6] Al-Mansour Z, Nelson BP, Evens AM (2013). Post-transplant lymphoproliferative disease (PTLD): risk factors, diagnosis, and current treatment strategies. Curr Hematol Malig Rep.

[7] Dharnidharka VR, Webster AC, Martinez OM, Preiksaitis JK, Leblond V, Choquet S (2016). Post-transplant lymphoproliferative disorders. Nat Rev Dis Primers.

[8] Green M, Michaels MG (2013). Epstein-Barr virus infection and posttransplant lymphoproliferative disorder. Am J Transplant.

[9] Molyneux EM, Rochford R, Griffin B (2012). Burkitt's lymphoma. Lancet.

[10] Opelz G, Döhler B (2004). Lymphomas after solid organ transplantation: a collaborative transplant study report. Am J Transplant.

[11] Morscio J, Dierickx D, Tousseyn T (2013). Molecular pathogenesis of B-cell posttransplant lymphoproliferative disorder: what do we know so far?. Clin Dev Immunol.

[12] Swerdlow SH, Campo E, Pileri SA (2016). The 2016 revision of the World Health Organization classification of lymphoid neoplasms. Blood.

[13] Bakker NA, van Imhoff GW, Verschuuren EA (2005). Early onset post-transplant lymphoproliferative disease is associated with allograft localization. Clin Transplant.

[14] Styczynski J, Gil L, Tridello G (2013). Response to rituximab-based therapy and risk factor analysis in Epstein Barr virus-related lymphoproliferative disorder after hematopoietic stem cell transplant in children and adults: a study from the infectious diseases working party of the Europea. Clin Infect Dis.

[15] Dunleavy K, Pittaluga S, Shovlin M (2013). Low-intensity therapy in adults with Burkitt's lymphoma. N Engl J Med.

[16] Cairo MS, Bishop M (2004). Tumour lysis syndrome: new therapeutic strategies and classification. Br J Haematol.

[17] Trappe R, Oertel S, Leblond V (2012). Sequential treatment with rituximab followed by CHOP chemotherapy in adult B-cell post-transplant lymphoproliferative disorder (PTLD): the prospective international multicentre phase 2 PTLD-1 trial. Lancet Oncol.

[18] Hill QA, Owen RG (2006). CNS prophylaxis in lymphoma: who to target and what therapy to use. Blood Rev.

[19] Ghobrial IM, Habermann TM, Maurer MJ (2005). Prognostic analysis for survival in adult solid organ transplant recipients with post-transplantation lymphoproliferative disorders. J Clin Oncol.

[20] Koffman BH, Kennedy AS, Heyman M (2000). Use of radiation therapy in posttransplant lymphoproliferative disorder (PTLD) after liver transplantation. Int J Cancer.

